# Maximum Varma Entropy Distribution with Conditional Value at Risk Constraints

**DOI:** 10.3390/e22060663

**Published:** 2020-06-16

**Authors:** Chang Liu, Chuo Chang, Zhe Chang

**Affiliations:** 1 Institute of Chinese Finance Studies, Southwestern University of Finance and Economics, Chengdu 611130, China; liuchang65@smail.swufe.edu.cn; 2 PBC School of Finance, Tsinghua University, Beijing 100083, China; changch.16@pbcsf.tsinghua.edu.cn; 3 Institute of High Energy Physics, Chinese Academy of Sciences, Beijing 100049, China; 4 University of Chinese Academy of Sciences, Beijing 100049, China

**Keywords:** maximum entropy, value at risk, portfolio selection, Varma entropy, investment risk, power law

## Abstract

It is well known that Markowitz’s mean-variance model is the pioneer portfolio selection model. The mean-variance model assumes that the probability density distribution of returns is normal. However, empirical observations on financial markets show that the tails of the distribution decay slower than the log-normal distribution. The distribution shows a power law at tail. The variance of a portfolio may also be a random variable. In recent years, the maximum entropy method has been widely used to investigate the distribution of return of portfolios. However, the mean and variance constraints were still used to obtain Lagrangian multipliers. In this paper, we use Conditional Value at Risk constraints instead of the variance constraint to maximize the entropy of portfolios. Value at Risk is a financial metric that estimates the risk of an investment. Value at Risk measures the level of financial risk within a portfolio. The metric is most commonly used by investment bank to determine the extent and occurrence ratio of potential losses in portfolios. Value at Risk is a single number that indicates the extent of risk in a given portfolio. This makes the risk management relatively simple. The Value at Risk is widely used in investment bank and commercial bank. It has already become an accepted standard in buying and selling assets. We show that the maximum entropy distribution with Conditional Value at Risk constraints is a power law. Algebraic relations between the Lagrangian multipliers and Value at Risk constraints are presented explicitly. The Lagrangian multipliers can be fixed exactly by the Conditional Value at Risk constraints.

## 1. Introduction

Markowitz’s mean-variance model [1] is the pioneer portfolio selection model. The mean-variance model is based on the assumption that return of assets follows a normal distribution. However, empirical observations on financial markets show that the tails of the distribution decay slower than the log-normal distribution (see [2,3,4]). A variety of models have been proposed to improve Markowitz’s mean-variance model. There is empirical evidence indicating that volatility is driven by a mean-reverting stochastic process (see [5,6,7,8,9]). It has been suggested that the volatility of an empirical financial market should be a stochastic quantity (see [10,11,12,13,14,15,16,17,18,19]). The autoregressive conditional heteroscedasticity (ARCH) model (see [20]) describes the variance of the current error term or innovation as a function of the actual sizes of the previous time periods’ error terms. If an autoregressive moving average model is assumed for the error variance, the model is a generalized autoregressive conditional heteroscedasticity (GARCH) model (see [8,9,21,22,23,24]). GARCH models are commonly employed in modeling financial time series that exhibit time-varying volatility and volatility clustering.

In particular, the power-law tails of empirical observations on financial markets have been studied extensively ([25,26,27,28,29]). Mandelbrot [25] first noticed the scaling properties of financial markets. It has been shown that the price distribution of financial assets is of the form of power law. Therefore, for distributions that are asymmetrical or non-normal, a different measure of uncertainty is required, which should be more dynamic and general than the variance, and does not rely on a specific distribution.

As entropy is a well-known measure of diversity, many scholars apply it to the portfolio selection and asset pricing. Philippatos and Wilson first used the concept of entropy to portfolio selection [30]. They tried to maximize the expected portfolio return and minimize the portfolio entropy. The concepts of individual entropy, joint entropy, and conditional entropy were introduced. The mean-entropy portfolios are consistent with the full covariance and the single-index models. Many progresses have been made in exploring entropy of the portfolio weights as a maximization objective to encourage diversification levels ([31,32,33,34,35,36,37]). Lassance [38] used the R$\overset{´}{e}$nyi entropy discussed the portfolio optimization. For a detailed review of the application of entropy, please see [39].

In this paper, following [40], we try to use Value at Risk constraints instead of the variance constraint to maximize the entropy of portfolios. Value at Risk is a financial metric that estimates the risk of an investment. Value at Risk measures the level of financial risk within a portfolio. It is the loss, which is exceeded with a given probability α, over a given horizon. For a finite horizon [0,T], we define W(0) as the initial wealth, and W(T) as the terminal-horizon wealth. If the value at risk horizon coincides with the investment horizon, we can describe the value at risk as [41,42]

(1) P(W(0)−W(T))≤VaR(α)≡1−α,α∈[0,1].

Thus, value at risk is the worse loss over a given time interval. The convenient way of management on value at risk is setting the VaR(α) to be maintained below a pre-specified level,

(2) VaR(α)≤W(0)−W,

where W is specified exogenously. Then, the value at risk constraint can be expressed as

(3) P(W(T)≥W)≥1−α.

The metric is most commonly used by an investment bank to determine the extent and occurrence ratio of potential losses in portfolios. Value at Risk is a single number that indicates the extent of risk in a given portfolio. This makes the risk management relatively simple. The Value at Risk is widely used in investment banks and commercial banks. It has already become an accepted standard in buying and selling assets. It is noticed that, in practice, financial agents control the magnitude of a loss rather than its probability. To control the magnitude of a loss, one should limit the expected shortfall,

(4) $$E[P\left(T\right)\left(W-W\left(T\right)\right)I_{\left\{W(T)\leq W\right\}}\leq \epsilon \;.$$

This constraint penalizes both a high probability of a loss and a high expected loss given there is a loss. It is a risk measure of time-*T* losses. This measure of risk is call the Conditional Value at Risk. It should be noticed that a generalized Markowitz’s mean-variance model with a Value at Risk constraint has been investigated for a long time [43,44]. However, the probability density distribution of returns was still assumed to be normal. In this paper, we show that the maximum entropy distribution with Value at Risk constraints is a power law. Algebraic relations between the Lagrangian multipliers and Value at Risk constraints are presented explicitly. The Lagrangian multipliers can be fixed exactly by the Value at Risk constraints.

## 2. Review on Maximum Entropy Distribution in Portfolio Selection

### 2.1. The Shannon Entropy

In a financial market, return *R* of a portfolio is a discrete random variable with probability $p_{i}$ (i=1,2,⋯,n). The portfolio with return $R_{i}$ constitutes a complete system of mutually exclusive events $E_{1}$, $E_{1}$, …, $E_{n}$ with probabilities $p_{1}$, $p_{2}$, …., $p_{n}$, respectively. Entropies are measures of uncertainty for such a system. We can define the Shannon entropy as

(5) $$H_{n}\left(p_{1},p_{2},\cdots ,p_{n}\right)=-\sum_{i=1}^{n}p_{i}\ln p_{i}\;,$$

with constraint $\sum_{i=1}^{n}p_{i}=1$.

If all probability $p_{i}$ with return $R_{i}$ are equal, $p_{i}=\frac{1}{n}$. At the case, the Shannon entropy is maximal. Shannon entropy satisfies the Gibbs inequality: $H_{n}\left(p_{1},p_{2},\cdots ,p_{n}\right)\leq \ln n$. It is monotonically increasing with *n*,

(6) $$H_{n}\left(p_{1},p_{2},\cdots ,p_{n}\right)\leq H_{n}\left(\frac{1}{n},\frac{1}{n},\cdots ,\frac{1}{n}\right)\leq H_{n+1}\left(\frac{1}{n+1},\frac{1}{n+1},\cdots ,\frac{1}{n+1}\right)\;.$$

The Shannon entropy is symmetric under permutation of any two variables $p_{j}$ and $p_{k}$. Enlarging by an event of probability 0 does not change the Shannon entropy. This is to say that the entropy is expansible,

(7) $$H_{n+1}\left(p_{1},p_{2},\cdots ,p_{n},0\right)=H_{n}\left(p_{1},p_{2},\cdots ,p_{n},0\right)\;.$$

If the portfolio *S* has return $R_{i}^{S}$ with probabilities $p_{i}\left(R_{i}^{S}\right)$ (i=1,2,⋯,n) and the portfolio *T* has return $R_{j}^{T}$ with probabilities $p_{j}\left(R_{j}^{T}\right)$ (j=1,2,⋯,m), then the probability of the portfolio S⊕T with return $R_{i}^{S}+R_{j}^{S}$ is $p_{ij}\left(R_{i}^{S}+R_{j}^{S}\right)=p\left(R_{i}^{S}\cap R_{j}^{T}\right)$ (i=1,2,⋯,n and j=1,2,⋯,m). It is not difficult to prove that

(8) H(S⊕T)≤H(S)+H(T).

Information expected from two portfolios is not greater than the sum of information expected from the single portfolio. It is called subadditivity.

If the two portfolios are independent, we have $p_{ij}\left(R_{i}^{S}+R_{j}^{S}\right)=p\left(R_{i}^{S}\right)p\left(R_{j}^{T}\right)$ (i=1,2,⋯,n and j=1,2,⋯,m). In this case, one has

(9) H(S⊕T)=H(S)+H(T).

Information expected from two independent portfolios equals the sum of informations expected from the single portfolio.This is called additivity.

Another characterization of the Shannon entropy is recursivity,

(10) $$H_{n}\left(p_{1},p_{2},p_{3},\cdots ,p_{n}\right)=H_{n-1}\left(p_{1}+p_{2},p_{3},\cdots ,p_{n}\right)+\left(p_{1}+p_{2}\right)H_{2}\left(\frac{p_{1}}{p_{1}+p_{2}},\frac{p_{2}}{p_{1}+p_{2}}\right)\;.$$

When dealing with continuous probability density distribution p(x), we can define the Shannon entropy as

(11) $$H\left(p\right)=-\int_{-\infty }^{\infty }p\left(x\right)\ln p\left(x\right)dx\;,$$

with constraint

(12) $$\int_{-\infty }^{\infty }p\left(x\right)dx=1\;,\;\;\;\;\;p\left(x\right)\geq 0\;.$$

Furthermore, one can set the mean and variance of the portfolio

(13) $$\int_{-\infty }^{\infty }xp\left(x\right)dx=\mu \;,\;\;\;\;\;\;\int_{-\infty }^{\infty }x^{2}p\left(x\right)dx=\sigma \;,$$

as constraints to probability density distribution of return p(x).

To maximize the Shannon entropy (11), we construct the Lagrangian

(14) $$\begin{array}{cc} L^{Shannon}= & -\int_{-\infty }^{\infty }p\left(x\right)\ln p\left(x\right)dx-\lambda \left[\int_{-\infty }^{\infty }p\left(x\right)dx-1\right]\\ & -\gamma_{1}\left[\int_{-\infty }^{\infty }xp\left(x\right)dx-\mu \right]-\gamma_{2}\left[\int_{-\infty }^{\infty }x^{2}p\left(x\right)dx-\sigma \right]\;. \end{array}$$

Lagrangian will be maximized when its functional variation with respect to the unknown probability density distribution p(x) is zero,

(15) $$\begin{array}{cc} \delta L^{Shannon} & =-\int_{-\infty }^{\infty }\left[\ln p\left(x\right)+1+\lambda +\gamma_{1}x+\gamma_{2}x^{2}\right]\delta p\left(x\right)dx\\ & =0\;. \end{array}$$

This leads immediately to

(16) $$p\left(x\right)=e^{-1-\lambda -\gamma_{1}x-\gamma_{2}x^{2}}\;.$$

The expressions of unity of probability density distribution, mean, and variance of portfolio,

(17) $$\begin{array}{c} \int_{-\infty }^{\infty }e^{\left(-1-\lambda -\gamma_{1}x-\gamma_{2}x^{2}\right)}dx=1\;,\\ \int_{-\infty }^{\infty }xe^{\left(-1-\lambda -\gamma_{1}x-\gamma_{2}x^{2}\right)}dx=\mu \;,\\ \int_{-\infty }^{\infty }x^{2}e^{\left(-1-\lambda -\gamma_{1}x-\gamma_{2}x^{2}\right)}dx=\sigma \;, \end{array}$$

can be integrated out and solved to fix the value of multiplier λ, $\gamma_{1}$, and $\gamma_{2}$. Thus, we get the maximum Shannon entropy distribution as

(18) $$p\left(x\right)=\frac{1}{\sqrt{2\pi }\sigma }\exp\left(-\frac{{\left(x-\mu \right)}^{2}}{2\sigma^{2}}\right)\;.$$

It is just the normal distribution.

### 2.2. The R$\overset{´}{e}$nyi Entropy

The R$\overset{´}{e}$nyi entropy of order α (≥0,≠1) is defined as

(19) $$H_{\alpha n}\left(p_{1},p_{2},\cdots ,p_{n}\right)=\frac{1}{1-\alpha }\ln\sum_{i=1}^{n}p_{i}^{\alpha }\;.$$

Notice a simple differential $\frac{da^{t}}{dt}=a^{t}\ln a$. In the limit of α→1, the R$\overset{´}{e}$nyi entropy behaves as

(20) $$\begin{array}{cc} \lim_{\alpha \to 1}H_{\alpha n}\left(p_{1},p_{2},\cdots ,p_{n}\right) & =\lim_{\alpha \to 1}\frac{1}{\frac{d(1-\alpha )}{d\alpha }}\frac{d}{d\alpha }\ln\sum_{i=1}^{n}p_{i}^{\alpha }\\ & =-\lim_{\alpha \to 1}\frac{\sum_{i=1}^{n}p_{i}^{\alpha }\ln p_{i}}{\sum_{i=1}^{n}p_{i}^{\alpha }}\\ & =-\sum_{i=1}^{n}p_{i}\ln p_{i}\;, \end{array}$$

where we have used L’H$\hat{o}$pital’s rule in the first equal and the constraint $\sum_{i=1}^{n}p_{i}=1$ at the third equal. Thus, in the limit of α→1, the R$\overset{´}{e}$nyi entropy reduces to the Shannon entropy. The R$\overset{´}{e}$nyi entropy is a generalization of the Shannon entropy.

The R$\overset{´}{e}$nyi entropy has similar properties to the Shannon entropy. It is additive. It has maximum = ln(n) for $p_{i}=\frac{1}{n}$. However, the R$\overset{´}{e}$nyi entropy contains additional parameter α which can be used to make it more or less sensitive to the shape of probability density distributions.

When dealing with continuous probability density distribution p(x), we can define the R$\overset{´}{e}$nyi entropy as

(21) $$H_{\alpha }\left(p\right)=\frac{1}{1-\alpha }\ln\int_{-\infty }^{\infty }p^{\alpha }\left(x\right)dx\;,$$

with constraint

(22) $$\int_{-\infty }^{\infty }p\left(x\right)dx=1\;,\;\;\;\;\;p\left(x\right)\geq 0\;.$$

Furthermore, one can set the mean and variance of the portfolio

(23) $$\int_{-\infty }^{\infty }xp\left(x\right)dx=\mu \;,\;\;\;\;\;\;\int_{-\infty }^{\infty }x^{2}p\left(x\right)dx=\sigma \;,$$

as constraints to probability density distribution of return p(x).

To maximize the R$\overset{´}{e}$nyi entropy (21), we construct the Lagrangian

(24) $$\begin{array}{cc} L^{Renyi}= & \frac{1}{1-\alpha }\ln\int_{-\infty }^{\infty }p^{\alpha }\left(x\right)dx-\lambda \left[\int_{-\infty }^{\infty }p\left(x\right)dx-1\right]\\ & -\gamma_{1}\left[\int_{-\infty }^{\infty }xp\left(x\right)dx-\mu \right]-\gamma_{2}\left[\int_{-\infty }^{\infty }x^{2}p\left(x\right)dx-\sigma \right]\;. \end{array}$$

Lagrangian will be maximized when its functional variation with respect to the unknown probability density distribution p(x) is zero,

(25) $$\begin{array}{cc} \delta L^{Renyi} & =\int_{-\infty }^{\infty }\left[\frac{\alpha }{1-\alpha }\frac{p^{\alpha -1}\left(x\right)}{\int_{-\infty }^{\infty }p^{\alpha }\left(x\right)dx}-\lambda -\gamma_{1}x-\gamma_{2}x^{2}\right]\delta p\left(x\right)dx\\ & =0\;. \end{array}$$

This leads immediately to

(26) $$p\left(x\right)=\frac{1}{H}{\left(\lambda +\gamma_{1}x+\gamma_{2}x^{2}\right)}^{\frac{1}{1-\alpha }}\;,$$

where we have used the notation $H={\left[\frac{1-\alpha }{\alpha }\int_{-\infty }^{\infty }p^{\alpha }\left(x\right)dx\right]}^{\frac{1}{\alpha -1}}$.

Furthermore, we can rewrite the probability density distribution as the form

(27) $$p\left(x\right)={\left(\bar{\lambda }+{\bar{\gamma }}_{1}x+{\bar{\gamma }}_{2}x^{2}\right)}^{\frac{1}{1-\alpha }}\;,$$

where we have introduced the notations $\bar{\lambda }\equiv \frac{\lambda }{H^{\alpha -1}}$, ${\bar{\gamma }}_{1}\equiv \frac{\gamma_{1}}{H^{\alpha -1}}$, ${\bar{\gamma }}_{2}\equiv \frac{\gamma_{2}}{H^{\alpha -1}}$. The parameters $\bar{\lambda }$, ${\bar{\gamma }}_{1}$, and ${\bar{\gamma }}_{2}$ can be fixed by the unity of probability density distribution, mean, and variance of portfolio.

## 3. Value at Risk

Value at Risk (VaR) is a financial metric that estimates the risk of an investment. VaR measures the level of financial risk within a portfolio. The metric is most commonly used by investment bank to determine the extent and occurrence ratio of potential losses in portfolios. VaR gives the probability of losing more than a given amount in a given portfolio. We measure VaR by assessing the amount of potential loss, the probability of occurrence for the amount of loss. Value at Risk is a single number that indicates the extent of risk in a given portfolio. Value at Risk is measured in either price units or as a percentage. This makes the risk management relatively simple. The Value at Risk is widely used in investment banks and commercial banks. It already has become an accepted standard in buying and selling assets.

One can use a historical method to calculate the VaR for a portfolio. The advantage of the historical method is simple. We take market data for the last few days to calculate the percentage change for each risk factor on each day. In addition, then calculate each percentage change with current market values to present scenarios for future value. For each of the scenarios, the portfolio is valued using pricing models. The shortcoming of the historical method is that empirical return of a portfolio is a stochastic variable and can not be obtained by historical data. That is to say, in principle, one can not use historical distribution of return to calculate future distribution.

To overcome the shortcoming of the historical method, one can use the parametric method to calculate the VaR for a portfolio. The present used parametric method is in fact a variance–covariance method. The parametric method assumes that the probability density distribution of return is a normal distribution. To calculate the VaR for a portfolio, one has to estimate the expected return and standard deviation of the return. If the distribution is almost normal and the mean and variance can be estimated reliably, the parametric method is best suited to risk measurement. However, it is well known that the empirical distribution of return of portfolios is fat tailed. It is almost a power law at tail.

The Monte Carlo Method has been proposed to deal with the problem met in the parameter model. One can calculate Value at Risk by randomly creating a set of scenarios for future returns using pricing models to estimate the change in value for each scenario. The Monte Carlo Method also assumes that there is a known probability distribution for risk factors. In fact, we do not have the exact distribution.

A useful alternative of the VaR is the Conditional Value at Risk (CVaR). CVaR is also known as the expected shortfall, average value at risk, tail VaR, mean excess loss, or mean shortfall. CVaR can be used to calculate the average of the losses that occur beyond the Value at Risk point in a distribution. In this paper, we should use maximum entropy to get the distribution.

## 4. Entropy Maximisation with VaR Constraints

A well-known generalization of Shannon’s entropy is Varma’s entropy. This kind of entropy was introduced by Varma [45]. Varma entropy plays an important role as a measure of complexity and uncertainty in different areas. Malhotra, Srivastava, and Taneja used Varma entropy to calibrate the risk-neutral density function [46]. In this section, we follow the method developed in [46].

Let p(x) be the unknown probability density distribution with normalization condition,

(28) $$\int_{-\infty }^{\infty }p\left(x\right)dx=1\;.$$

The two parameters Varma entropy would be defined as

(29) $$H_{\alpha \beta }=\frac{1}{\beta -\alpha }\ln\int_{-\infty }^{\infty }p^{\alpha +\beta -1}\left(x\right)dx\;.$$

The global mean constraint is of the form

(30) $$E\left(x\right)=\int_{-\infty }^{\infty }xp\left(x\right)dx=\mu \;.$$

In value at risk management, we have the constraints of tail probability and expected shortfall (CVaR). The tail probability is defined as

(31) $$P\left(x\leq K\right)=\int_{-\infty }^{K}p\left(x\right)dx=\epsilon \;.$$

The expected shortfall can be expressed as

(32) $$E\left(x|x\leq K\right)=\nu_{-}\;,$$

or

(33) $$E\left(xI_{x\leq K}\right)=\int_{-\infty }^{K}xp\left(x\right)dx=\epsilon \nu_{-}\;.$$

We can rewrite formally the tail probability and expected shortfall as follows:

(34) $$\left\{\begin{array}{ccc} P(x\leq K) & = & \int_{-\infty }^{\infty }g^{Tail}\left(x\right)p\left(x\right)dx=\epsilon \;,\\ g^{Tail}\left(x\right) & = & \left\{\begin{array}{c} 1\;,\;\;\;\;x\leq K\;,\\ 0\;,\;\;\;\;x>K\;, \end{array}\right. \end{array}\right.$$

and

(35) $$\left\{\begin{array}{ccc} E(xI_{x\leq K}) & = & \int_{-\infty }^{\infty }g^{CVaR}\left(x\right)p\left(x\right)dx=\epsilon \nu_{-}\;,\\ g^{CVaR}\left(x\right) & = & \left\{\begin{array}{c} x\;,\;\;\;\;x\leq K\;,\\ 0\;,\;\;\;\;x>K\;. \end{array}\right. \end{array}\right.$$

Jaynes’ maximum entropy principle states that, out of all possible distributions consistent with the constraints, the optimal one has the maximum uncertainty. It is least committed to the information not given to us. In other words, the optimal distribution is most random and most unbiased. To maximize the Varma entropy (29), we construct the Lagrangian

(36) $$\begin{array}{ccc} L & = & \frac{1}{\beta -\alpha }\ln\int_{-\infty }^{\infty }p^{\alpha +\beta -1}\left(x\right)dx-\lambda_{0}\left(\int_{-\infty }^{\infty }p\left(x\right)dx-1\right)\\ \; & \; & -\lambda_{1}\left(\int_{-\infty }^{\infty }xp\left(x\right)dx-\mu \right)-\gamma^{Tail}\left(\int_{-\infty }^{\infty }g^{Tail}\left(x\right)p\left(x\right)dx-\epsilon \right)\\ \; & \; & -\gamma^{CVaR}\left(\int_{-\infty }^{\infty }g^{CVaR}\left(x\right)p\left(x\right)dx-\epsilon \nu_{-}\right)\;. \end{array}$$

The Lagrangian will be maximized when its functional variation with respect to the unknown probability density distribution p(x) is zero,

(37) $$\begin{array}{ccc} \delta L & = & \int_{-\infty }^{\infty }\left[\frac{\alpha +\beta -1}{\beta -\alpha }\frac{p^{\alpha +\beta -2}\left(x\right)}{\int_{-\infty }^{\infty }p^{\alpha +\beta -1}\left(x\right)dx}-\lambda_{0}-\lambda_{1}x-\gamma^{Tail}g^{Tail}\left(x\right)-\gamma^{CVaR}g^{CVaR}\left(x\right)\right]\delta p\left(x\right)\\ \; & = & 0\;. \end{array}$$

This leads immediately to

(38) $$\frac{\alpha +\beta -1}{\beta -\alpha }\frac{p^{\alpha +\beta -2}\left(x\right)}{\int_{-\infty }^{\infty }p^{\alpha +\beta -1}\left(x\right)dx}-\lambda_{0}-\lambda_{1}x-\gamma^{Tail}g^{Tail}\left(x\right)-\gamma^{CVaR}g^{CVaR}\left(x\right)=0\;.$$

Thus, we obtain the probability density distribution under the constraints of value at risk as

(39) $$p\left(x\right)={\left[\frac{\beta -\alpha }{\alpha +\beta -1}\int_{-\infty }^{\infty }p^{\alpha +\beta -1}\left(x\right)dx\cdot \left[\lambda_{0}+\lambda_{1}x+\gamma^{Tail}g^{Tail}\left(x\right)+\gamma^{CVaR}g^{CVaR}\left(x\right)\right]\right]}^{\frac{1}{\alpha +\beta -2}}\;.$$

We can rewrite the probability density distribution under the constraints of value at risk as

(40) $$p\left(x\right)=\frac{1}{N}{\left[\lambda_{0}+\lambda_{1}x+\gamma^{Tail}g^{Tail}\left(x\right)+\gamma^{CVaR}g^{CVaR}\left(x\right)\right]}^{\frac{1}{\alpha +\beta -2}}\;,$$

where

$$N={\left[\frac{\beta -\alpha }{\alpha +\beta -1}\int_{-\infty }^{\infty }p^{\alpha +\beta -1}\left(x\right)dx\right]}^{\frac{1}{2-\alpha -\beta }}$$

is the normalization factor.

Furthermore, we can rewrite the probability density distribution under the constraints of value at risk as the form

(41) $$p\left(x\right)={\left[{\bar{\lambda }}_{0}+{\bar{\lambda }}_{1}x+{\bar{\gamma }}^{Tail}g^{Tail}\left(x\right)+{\bar{\gamma }}^{CVaR}g^{CVaR}\left(x\right)\right]}^{\frac{1}{\alpha +\beta -2}}\;,$$

where we have introduced the notations ${\bar{\lambda }}_{0}\equiv \frac{\lambda_{0}}{N^{\alpha +\beta -2}}$, ${\bar{\lambda }}_{1}\equiv \frac{\lambda_{1}}{N^{\alpha +\beta -2}}$, ${\bar{\gamma }}^{Tail}\equiv \frac{\gamma^{Tail}}{N^{\alpha +\beta -2}}$, and ${\bar{\gamma }}^{CVaR}\equiv \frac{\gamma^{CVaR}}{N^{\alpha +\beta -2}}$.

## 5. Lagrangian Multipliers Determination

By making use of the probability density distribution under the constraints of value at risk (41), we can express the unity condition (28) as

(42) $$\int_{-\infty }^{K}{\left[{\bar{\lambda }}_{0}+{\bar{\lambda }}_{1}x+{\bar{\gamma }}^{Tail}+{\bar{\gamma }}^{CVaR}x\right]}^{\frac{1}{\alpha +\beta -2}}dx+\int_{K}^{\infty }{\left[{\bar{\lambda }}_{0}+{\bar{\lambda }}_{1}x\right]}^{\frac{1}{\alpha +\beta -2}}dx=1\;.$$

This constraint can be integrated out explicitly,

(43) $$\begin{array}{cc} \frac{1}{{\bar{\lambda }}_{1}+{\bar{\gamma }}^{CVaR}}\frac{\alpha +\beta -2}{\alpha +\beta -1}{\left.{\left[{\bar{\lambda }}_{0}+{\bar{\lambda }}_{1}x+{\bar{\gamma }}^{Tail}+{\bar{\gamma }}^{CVaR}x\right]}^{\frac{\alpha +\beta -1}{\alpha +\beta -2}}\right|}_{-\infty }^{K} & \\ +\frac{1}{{\bar{\lambda }}_{1}}\frac{\alpha +\beta -2}{\alpha +\beta -1}{\left.{\left[{\bar{\lambda }}_{0}+{\bar{\lambda }}_{1}x\right]}^{\frac{\alpha +\beta -1}{\alpha +\beta -2}}\right|}_{-\infty }^{K}=1 & . \end{array}$$

If the two parameters of the Varma entropy satisfy 1<α+β<2, the above integration equals zero at −∞ and *∞*. In addition, we have

(44) $$\begin{array}{cc} \frac{\alpha +\beta -2}{\alpha +\beta -1}\left[\frac{1}{{\bar{\lambda }}_{1}+{\bar{\gamma }}^{CVaR}}{\left[{\bar{\lambda }}_{0}+{\bar{\gamma }}^{Tail}+\left({\bar{\lambda }}_{1}+{\bar{\gamma }}^{CVaR}\right)K\right]}^{\frac{\alpha +\beta -1}{\alpha +\beta -2}}\right. & \\ \left.-\frac{1}{{\bar{\lambda }}_{1}}{\left[{\bar{\lambda }}_{0}+{\bar{\lambda }}_{1}K\right]}^{\frac{\alpha +\beta -1}{\alpha +\beta -2}}\right]=1 & . \end{array}$$

By making use of the probability density distribution under the constraints of value at risk (41), we can express the mean constraint (30) as

(45) $$\int_{-\infty }^{K}x{\left[{\bar{\lambda }}_{0}+{\bar{\lambda }}_{1}x+{\bar{\gamma }}^{Tail}+{\bar{\gamma }}^{CVaR}x\right]}^{\frac{1}{\alpha +\beta -2}}dx+\int_{K}^{\infty }x{\left[{\bar{\lambda }}_{0}+{\bar{\lambda }}_{1}x\right]}^{\frac{1}{\alpha +\beta -2}}dx=\mu \;.$$

Using integration by parts, we have

(46) $$\begin{array}{c} \frac{1}{{\bar{\lambda }}_{1}+{\bar{\gamma }}^{CVaR}}\frac{\alpha +\beta -2}{\alpha +\beta -1}x{\left.{\left[{\bar{\lambda }}_{0}+{\bar{\lambda }}_{1}x+{\bar{\gamma }}^{Tail}+{\bar{\gamma }}^{CVaR}x\right]}^{\frac{\alpha +\beta -1}{\alpha +\beta -2}}\right|}_{-\infty }^{K}\\ \;\;\;\;\;\;-\int_{-\infty }^{K}\frac{1}{{\bar{\lambda }}_{1}+{\bar{\gamma }}^{CVaR}}\frac{\alpha +\beta -2}{\alpha +\beta -1}{\left[{\bar{\lambda }}_{0}+{\bar{\lambda }}_{1}x+{\bar{\gamma }}^{Tail}+{\bar{\gamma }}^{CVaR}x\right]}^{\frac{\alpha +\beta -1}{\alpha +\beta -2}}dx\\ \;\;\;\;\;+\frac{1}{{\bar{\lambda }}_{1}}\frac{\alpha +\beta -2}{\alpha +\beta -1}x{\left.{\left[{\bar{\lambda }}_{0}+{\bar{\lambda }}_{1}x\right]}^{\frac{\alpha +\beta -1}{\alpha +\beta -2}}\right|}_{K}^{\infty }\\ \;\;\;\;\;\;\;\;\;\;\;\;\;\;\;\;\;\;\;\;\;-\int_{K}^{\infty }\frac{1}{{\bar{\lambda }}_{1}}\frac{\alpha +\beta -2}{\alpha +\beta -1}{\left[{\bar{\lambda }}_{0}+{\bar{\lambda }}_{1}x\right]}^{\frac{\alpha +\beta -1}{\alpha +\beta -2}}dx=\mu \;. \end{array}$$

This constraint can be integrated out explicitly,

(47) $$\begin{array}{c} \frac{\alpha +\beta -2}{\alpha +\beta -1}\left\{\frac{1}{{\bar{\lambda }}_{1}+{\bar{\gamma }}^{CVaR}}x{\left.{\left[{\bar{\lambda }}_{0}+{\bar{\lambda }}_{1}x+{\bar{\gamma }}^{Tail}+{\bar{\gamma }}^{CVaR}x\right]}^{\frac{\alpha +\beta -1}{\alpha +\beta -2}}\right|}_{-\infty }^{K}\right.\\ \;\;-\frac{1}{{\left({\bar{\lambda }}_{1}+{\bar{\gamma }}^{CVaR}\right)}^{2}}\frac{\alpha +\beta -2}{2(\alpha +\beta )-3}{\left.{\left[{\bar{\lambda }}_{0}+{\bar{\lambda }}_{1}x+{\bar{\gamma }}^{Tail}+{\bar{\gamma }}^{CVaR}x\right]}^{\frac{2(\alpha +\beta )-3}{\alpha +\beta -2}}\right|}_{-\infty }^{K}\\ \;\;+\frac{1}{{\bar{\lambda }}_{1}}x{\left.{\left[{\bar{\lambda }}_{0}+{\bar{\lambda }}_{1}x\right]}^{\frac{\alpha +\beta -1}{\alpha +\beta -2}}\right|}_{K}^{\infty }\\ \;\;\;\;\;\;\;\;\;\;\;\;\;\;\;\;\;\;\;\;\;-\left.\frac{1}{{\bar{\lambda }}_{1}^{2}}\frac{\alpha +\beta -2}{2(\alpha +\beta )-3}{\left.{\left[{\bar{\lambda }}_{0}+{\bar{\lambda }}_{1}x\right]}^{\frac{2(\alpha +\beta )-3}{\alpha +\beta -2}}\right|}_{K}^{\infty }\right\}=\mu \;. \end{array}$$

If the two parameters of the Varma entropy satisfy $\frac{3}{2}<\alpha +\beta <2$, the above integration equals zero at −∞ and *∞*. In addition, we have

(48) $$\begin{array}{c} \frac{\alpha +\beta -2}{\alpha +\beta -1}\left\{K\left[\frac{1}{{\bar{\lambda }}_{1}+{\bar{\gamma }}^{CVaR}}{\left[{\bar{\lambda }}_{0}+{\bar{\gamma }}^{Tail}+\left({\bar{\lambda }}_{1}+{\bar{\gamma }}^{CVaR}\right)K\right]}^{\frac{\alpha +\beta -1}{\alpha +\beta -2}}-\frac{1}{{\bar{\lambda }}_{1}}{\left[{\bar{\lambda }}_{0}+{\bar{\lambda }}_{1}K\right]}^{\frac{\alpha +\beta -1}{\alpha +\beta -2}}\right]\right.\\ \;\;\;\;\;\;\;\;\;\;-\frac{\alpha +\beta -2}{2(\alpha +\beta )-3}\left[\frac{1}{{\left({\bar{\lambda }}_{1}+{\bar{\gamma }}^{CVaR}\right)}^{2}}{\left[{\bar{\lambda }}_{0}+{\bar{\gamma }}^{Tail}+\left({\bar{\lambda }}_{1}+{\bar{\gamma }}^{CVaR}\right)K\right]}^{\frac{2(\alpha +\beta )-3}{\alpha +\beta -2}}\right.\\ \;\;\;\;\;\;\;\;\;\;\;\;\;\;\;\;\;\;\;\;\;\;\;\;\;\;\;\;\;\;\left.\left.-\frac{1}{{\bar{\lambda }}_{1}^{2}}{\left[{\bar{\lambda }}_{0}+{\bar{\lambda }}_{1}K\right]}^{\frac{2(\alpha +\beta )-3}{\alpha +\beta -2}}\right]\right\}=\mu \;. \end{array}$$

By making use of the probability density distribution under the constraints of value at risk (41), we can express the tail probability constraint (31) as

(49) $$\int_{-\infty }^{K}{\left[{\bar{\lambda }}_{0}+{\bar{\lambda }}_{1}x+{\bar{\gamma }}^{Tail}+{\bar{\gamma }}^{CVaR}x\right]}^{\frac{1}{\alpha +\beta -2}}dx=\epsilon \;.$$

This constraint can be integrated out explicitly,

(50) $$\frac{1}{{\bar{\lambda }}_{1}+{\bar{\gamma }}^{CVaR}}\frac{\alpha +\beta -2}{\alpha +\beta -1}{\left.{\left[{\bar{\lambda }}_{0}+{\bar{\lambda }}_{1}x+{\bar{\gamma }}^{Tail}+{\bar{\gamma }}^{CVaR}x\right]}^{\frac{\alpha +\beta -1}{\alpha +\beta -2}}\right|}_{-\infty }^{K}=\epsilon \;.$$

If the two parameters of the Varma entropy satisfy 1<α+β<2, the above integration equals zero at −∞. In addition, we have

(51) $$\frac{\alpha +\beta -2}{\alpha +\beta -1}\frac{1}{{\bar{\lambda }}_{1}+{\bar{\gamma }}^{CVaR}}{\left[{\bar{\lambda }}_{0}+{\bar{\gamma }}^{Tail}+\left({\bar{\lambda }}_{1}+{\bar{\gamma }}^{CVaR}\right)K\right]}^{\frac{\alpha +\beta -1}{\alpha +\beta -2}}=\epsilon \;.$$

By making use of the probability density distribution under the constraints of value at risk (41), we can express the expected shortfall constraint (33) as

(52) $$\int_{-\infty }^{K}x{\left[{\bar{\lambda }}_{0}+{\bar{\lambda }}_{1}x+{\bar{\gamma }}^{Tail}+{\bar{\gamma }}^{CVaR}x\right]}^{\frac{1}{\alpha +\beta -2}}dx=\epsilon \nu_{-}\;.$$

Using integration by parts, we have

(53) $$\begin{array}{c} \frac{1}{{\bar{\lambda }}_{1}+{\bar{\gamma }}^{CVaR}}\frac{\alpha +\beta -2}{\alpha +\beta -1}x{\left.{\left[{\bar{\lambda }}_{0}+{\bar{\lambda }}_{1}x+{\bar{\gamma }}^{Tail}+{\bar{\gamma }}^{CVaR}x\right]}^{\frac{\alpha +\beta -1}{\alpha +\beta -2}}\right|}_{-\infty }^{K}\\ \;\;-\int_{-\infty }^{K}\frac{1}{{\bar{\lambda }}_{1}+{\bar{\gamma }}^{CVaR}}\frac{\alpha +\beta -2}{\alpha +\beta -1}{\left[{\bar{\lambda }}_{0}+{\bar{\lambda }}_{1}x+{\bar{\gamma }}^{Tail}+{\bar{\gamma }}^{CVaR}x\right]}^{\frac{\alpha +\beta -1}{\alpha +\beta -2}}dx=\epsilon \nu_{-}\;. \end{array}$$

This constraint can be integrated out explicitly,

(54) $$\begin{array}{c} \frac{\alpha +\beta -2}{\alpha +\beta -1}\left\{\frac{1}{{\bar{\lambda }}_{1}+{\bar{\gamma }}^{CVaR}}x{\left.{\left[{\bar{\lambda }}_{0}+{\bar{\lambda }}_{1}x+{\bar{\gamma }}^{Tail}+{\bar{\gamma }}^{CVaR}x\right]}^{\frac{\alpha +\beta -1}{\alpha +\beta -2}}\right|}_{-\infty }^{K}\right.\\ \;\;\left.-\frac{1}{{\left({\bar{\lambda }}_{1}+{\bar{\gamma }}^{CVaR}\right)}^{2}}\frac{\alpha +\beta -2}{2(\alpha +\beta )-3}{\left.{\left[{\bar{\lambda }}_{0}+{\bar{\lambda }}_{1}x+{\bar{\gamma }}^{Tail}+{\bar{\gamma }}^{CVaR}x\right]}^{\frac{2(\alpha +\beta )-3}{\alpha +\beta -2}}\right|}_{-\infty }^{K}\right\}=\epsilon \nu_{-}\;. \end{array}$$

If the two parameters of the Varma entropy satisfy $\frac{3}{2}<\alpha +\beta <2$, the above integration equals zero at −∞. In addition, we have

(55) $$\begin{array}{c} \frac{\alpha +\beta -2}{\alpha +\beta -1}\left\{\frac{1}{{\bar{\lambda }}_{1}+{\bar{\gamma }}^{CVaR}}K{\left[{\bar{\lambda }}_{0}+{\bar{\gamma }}^{Tail}+\left({\bar{\lambda }}_{1}++{\bar{\gamma }}^{CVaR}\right)K\right]}^{\frac{\alpha +\beta -1}{\alpha +\beta -2}}\right.\\ \;\;\;\;\;\left.-\frac{\alpha +\beta -2}{2(\alpha +\beta )-3}\frac{1}{{\left({\bar{\lambda }}_{1}+{\bar{\gamma }}^{CVaR}\right)}^{2}}{\left[{\bar{\lambda }}_{0}+{\bar{\gamma }}^{Tail}+\left({\bar{\lambda }}_{1}++{\bar{\gamma }}^{CVaR}\right)K\right]}^{\frac{2(\alpha +\beta )-3}{\alpha +\beta -2}}\right\}=\epsilon \nu_{-}\;. \end{array}$$

For given parameters of the Varma entropy α and β, as well as the value-at-risk parameters $\theta =(K,\epsilon ,\nu_{-})$, we can use the algebraic Equations (44), (48), (51), and (55) determining uniquely the parameters ${\bar{\lambda }}_{0}$, ${\bar{\lambda }}_{1}$, ${\bar{\gamma }}^{Tail}$, and ${\bar{\gamma }}^{CVaR}$ in the probability density distribution (41). It should be noticed that, to set up all of the algebraic Equations (44), (48), (51), and (55), we limited the given parameters of the Varma entropy as $\frac{3}{2}<\alpha +\beta <2$.

## 6. Maximum Varma Entropy Distribution

The constraint on parameters of the Varma entropy $\frac{3}{2}<\alpha +\beta <2$ can be utilized by setting $\alpha +\beta =\frac{2q-1}{q}$ with q≥2. In addition, then the probability density distribution under the constraints of value at risk (41) becomes

(56) $$p\left(x\right)=\frac{1}{{\left[{\bar{\lambda }}_{0}+{\bar{\lambda }}_{1}x+{\bar{\gamma }}^{Tail}g^{Tail}\left(x\right)+{\bar{\gamma }}^{CVaR}g^{CVaR}\left(x\right)\right]}^{q}}\;,$$

The constraint on distribution parameters (44) becomes

(57) $$\left[\frac{1}{{\bar{\lambda }}_{1}+{\bar{\gamma }}^{CVaR}}{\left[{\bar{\lambda }}_{0}+{\bar{\gamma }}^{Tail}+\left({\bar{\lambda }}_{1}+{\bar{\gamma }}^{CVaR}\right)K\right]}^{1-q}-\frac{1}{{\bar{\lambda }}_{1}}{\left[{\bar{\lambda }}_{0}+{\bar{\lambda }}_{1}K\right]}^{1-q}\right]=1-q\;.$$

The constraint on distribution parameters (48) becomes

(58) $$\begin{array}{c} K\left[\frac{1}{{\bar{\lambda }}_{1}+{\bar{\gamma }}^{CVaR}}{\left[{\bar{\lambda }}_{0}+{\bar{\gamma }}^{Tail}+\left({\bar{\lambda }}_{1}+{\bar{\gamma }}^{CVaR}\right)K\right]}^{1-q}-\frac{1}{{\bar{\lambda }}_{1}}{\left[{\bar{\lambda }}_{0}+{\bar{\lambda }}_{1}K\right]}^{1-q}\right]\\ \;\;\;\;\;\;\;\;\;\;-\frac{1}{2-q}\left[\frac{1}{{\left({\bar{\lambda }}_{1}+{\bar{\gamma }}^{CVaR}\right)}^{2}}{\left[{\bar{\lambda }}_{0}+{\bar{\gamma }}^{Tail}+\left({\bar{\lambda }}_{1}+{\bar{\gamma }}^{CVaR}\right)K\right]}^{2-q}\right.\\ \;\;\;\;\;\;\;\;\;\;\;\;\;\;\;\;\;\;\;\;\;\;\;\;\;\;\;\;\;\;\;\;\;\;\;\;\;\;\;\;\;\;\;\;\;\;\;\;\left.-\frac{1}{{\bar{\lambda }}_{1}^{2}}{\left[{\bar{\lambda }}_{0}+{\bar{\lambda }}_{1}K\right]}^{2-q}\right]=\left(1-q\right)\mu \;. \end{array}$$

The constraint on distribution parameters (51) becomes

(59) $$\frac{1}{{\bar{\lambda }}_{1}+{\bar{\gamma }}^{CVaR}}{\left[{\bar{\lambda }}_{0}+{\bar{\gamma }}^{Tail}+\left({\bar{\lambda }}_{1}+{\bar{\gamma }}^{CVaR}\right)K\right]}^{1-q}=\left(1-q\right)\epsilon \;.$$

The constraint on distribution parameters (55) becomes

(60) $$\begin{array}{c} \frac{1}{{\bar{\lambda }}_{1}+{\bar{\gamma }}^{CVaR}}K{\left[{\bar{\lambda }}_{0}+{\bar{\gamma }}^{Tail}+\left({\bar{\lambda }}_{1}++{\bar{\gamma }}^{CVaR}\right)K\right]}^{1-q}\\ \;\;\;\;\;-\frac{1}{2-q}\frac{1}{{\left({\bar{\lambda }}_{1}+{\bar{\gamma }}^{CVaR}\right)}^{2}}{\left[{\bar{\lambda }}_{0}+{\bar{\gamma }}^{Tail}+\left({\bar{\lambda }}_{1}++{\bar{\gamma }}^{CVaR}\right)K\right]}^{2-q}=\left(1-q\right)\epsilon \nu_{-}\;. \end{array}$$

Equations (57), (58), (59), and (60) constitute a complete algebraic relations for the parameters in the probability density distribution (56). It is not difficult to get a numerical solution [46]. Then, we obtain a probability density distribution of power law form, which satisfies the VaR constraints.

## 7. Discussion and Conclusions

In this paper, we used Conditional Value at Risk constraints instead of the variance constraint to maximize the entropy of portfolios. It has been noticed that Value at Risk is a financial metric that estimates the risk of an investment. Value at Risk measures the level of financial risk within a portfolio. The metric is most commonly used by investment banks to determine the extent and occurrence ratio of potential losses in portfolios. Value at Risk is a single number that indicates the extent of risk in a given portfolio. This makes the risk management relatively simple. The Value at Risk is widely used in investment banks and commercial banks. It has already become an accepted standard in buying and selling assets. We show that the maximum entropy distribution with Conditional Value at Risk constraints is a power law. Algebraic relations between the Lagrangian multipliers and Value at Risk constraints are presented explicitly. The Lagrangian multipliers can be fixed exactly by the Conditional Value at Risk constraints. Empirical observations on financial markets show really that the tails of the distribution decay slower than the log-normal distribution. In particular, the power-law tails of empirical observations on financial markets have been studied extensively. By using the maximum Varma entropy with CVaR constraints, we obtained a proper probability density distribution of returns.

Recently, there has been a surge of interest in risk management involving more sophistical distortion measures [47,48,49]. The distortion risk measure encompasses VaR and CVaR as its special cases. Using a distortion function *g*, we can express the distortion risk measure $\rho^{g}$ as

(61) $$\rho^{g}\left(X\right)=\int_{0}^{1}VaR\left(X\right)d\tilde{g}\left(s\right),$$

where $\tilde{g}\left(s\right)=1-g\left(1-s\right)$. It is interesting to discuss the maximum entropy distribution with distortion risk measure constraint. To obtain a distribution, we need know the specified form of the distortion function. For example, the mean-CVaR distortion risk measure is defined as [50]

(62) $$\rho \left(X\right)=\frac{1+\theta }{1+\beta }\left[E\left(X\right)+\beta CVaR_{\alpha }\left(X\right)\right]\;,$$

where θ, β≥0 and α∈[0,1). We can redefine the $g^{CVaR}\left(x\right)$ in (35) as

(63) $$g^{MCVaR}\left(x\right)=\left\{\begin{array}{c} (1+\beta )x\;,\;\;\;\;x\leq K\;,\\ x\;,\;\;\;\;x>K\;. \end{array}\right.$$

In addition, all of calculations can be mimicked straightforwardly. One also gets a power law distribution. Here, we thank the anonymous reviewer for suggesting to us to notice the distortion risk measure.
